# Nutritional, Thermal, and Energetic Characterization of Two Morphotypes of Andean Mashua (*Tropaeolum tuberosum* Ruiz & Pavón) Flours from Peru

**DOI:** 10.3390/molecules30173560

**Published:** 2025-08-30

**Authors:** Gilmar Peña-Rojas, Vidalina Andía-Ayme, Alberto Fernández-Torres, Juan Z. Dávalos-Prado, Oscar Herrera-Calderon

**Affiliations:** 1 Laboratorio de Biología Celular y Molecular, Universidad Nacional de San Cristóbal de Huamanga, Ayacucho 05003, Peru; 2 Laboratory of Food Microbiology, Biological Sciences Faculty, Universidad Nacional de San Cristóbal de Huamanga, Ayacucho 05003, Peru; vidalina.andia@unsch.edu.pe; 3 Instituto Ciencia y Tecnología de Polímeros-CSIC, Juan de la Cierva 3, 28006 Madrid, Spain; a.f.torres@csic.es; 4 Instituto de Química-Física “Blas Cabrera”-CSIC, Serrano 119, 28006 Madrid, Spain; jdavalos@iqf.csic.es; 5 Department of Pharmacology, Bromatology and Toxicology, Faculty of Pharmacy and Biochemistry, Universidad Nacional Mayor de San Marcos, Lima 15001, Peru

**Keywords:** mashua flour, thermal analysis, net calorific value, elemental composition, functional food ingredients

## Abstract

*Tropaeolum tuberosum* (mashua) is a native Andean tuber recognized for its high nutritional and bioactive compound content. Among the various morphotypes, the black and yellow variants show potential differences in composition and functionality. This study aimed to compare the thermo-energetic, nutritional, and physicochemical characteristics of two morphotypes (black and yellow) of *Tropaeolum tuberosum* flour from the Peruvian Andes. Flours were obtained from tubers harvested in Ayacucho, Peru, and analyzed using elemental analysis for carbon, hydrogen, nitrogen, and sulfur (CHNS), inductively coupled plasma optical emission spectrometry (ICP-OES), scanning electron microscopy (SEM), differential scanning calorimetry (DSC), thermogravimetric analysis (TGA), and bomb calorimetry. The empirical formula is CH_1.74_O_0.91_N_0.06_S_0.005_ for black mashua and CH_1.78_O_0.92_N_0.05_S_0.005_ for yellow mashua. Black flour exhibited higher protein (17.6% vs. 14.8%) and fat contents (8.0% vs. 6.7%), along with nearly double the iron content. Both flours showed similar starch granule morphology and gelatinization enthalpy (~2 J/g), but the black flour had higher gelatinization temperatures. Calorimetric analysis revealed a greater net calorific value (qNCV) in black mashua flour (4157 ± 22 kcal/kg) than in yellow flour (4022 ± 19 kcal/kg). The thermogravimetric profiles indicated good thermal stability with approximately 30% residual mass. These findings suggested that black mashua flour possesses superior nutritional and energy characteristics, supporting its application in functional food formulations and energy-rich gluten-free products.

## 1. Introduction

The Andean tuber *Tropaeolum tuberosum* Ruiz & Pavón, commonly known as mashua, is a native crop from Peru, Bolivia, Argentina Northwest, Ecuador and Colombia, and typically grows between 1500 and 4300 m above sea level in the Andean region [1]. Mashua flour has garnered increasing attention because of its nutritional and techno-functional attributes. One study has demonstrated its efficacy as an ingredient in processed foods, such as sausage formulations, where it exhibits favorable water retention and functional properties [2]. Starches extracted from mashua display higher viscosity and stabilizing capacity than potato starch, indicating their potential as cost-effective thickening agents [3]. Furthermore, recent characterization of mashua genotypes has revealed elevated levels of protein, minerals, and bioactive compounds, suggesting its application as a functional and nutrient-rich flour [4]. Comparative analyses of Andean tuber flours suggest that mashua contributes significantly to protein content and digestible starch, positioning it as a value-added ingredient for gluten-free and gluten-enriched products [5]. Its starch profile also imparts desirable pasting and textural properties, which are advantageous for food applications that require heat stability and viscosity control [6].

Traditionally valued for its edible tubers, mashua has recently garnered attention because of its exceptional nutritional and medicinal properties, including its high levels of glucosinolates, polyphenols, anthocyanins, and other bioactive compounds [7]. Mashua has been used to treat various ailments such as liver and kidney diseases, venereal diseases, skin ulcers, parasites, heart diseases, anaphrodisiac and prostate diseases. Regarding the specific uses based on tuber color, black tubers are used for treating gonorrhea and scalp infections whereas yellow tubers are used against urinary and prostate diseases [8].

Mashua tubers exhibit a range of morphotypes which are identifiable by the color of their skin and flesh, including black, purple, red, and yellow [9]. Previous research has suggested that darker morphotypes generally contain higher levels of antioxidants and glucosinolates than lighter morphotypes [10]. Despite the increasing interest in this area, there is still a scarcity of studies focusing on the physicochemical and thermal properties of mashua, especially processed forms such as flour. Conversely, flours made from Andean tubers are increasingly being investigated as components of functional and gluten-free food products [5]. Therefore, it is crucial to understand their thermal properties, such as gelatinization and thermal degradation, as well as their energy content, including their net calorific value. These attributes are important not only for food processing and stability but also for assessing their potential for high-energy nutritional use [11].

In recent years, there has been growing interest in native Andean flours as a sustainable alternative to conventional cereal-based ingredients [12]. These flours, derived from underutilized crops such as mashua, oca (*Oxalis tuberosa*), and ulluco (*Ullucus tuberosus*), offer gluten-free, nutrient-dense profiles suitable for specialized diets and functional food development [3,6,13]. However, their physicochemical behavior under thermal processing conditions remains poorly understood, limiting their industrial application. A detailed evaluation of the thermal transitions, degradation stability, and energy release upon combustion is particularly relevant for optimizing their use in baking, extrusion, and dehydration processes [14]. Moreover, these properties may be useful in nutritionally dense emergency foods or bioenergy substrates.

The comprehensive evaluation of the thermo-energetic, nutritional, and physicochemical parameters of *Tropaeolum tuberosum* flours will provide valuable information for their application in food formulations. Specifically, these data can inform recipe development and the optimization of processing conditions for gluten-free bakery, snack, and pasta products. Given their natural starch content, distinctive bioactive profile, and absence of gluten, these flours represent a promising ingredient for enhancing both the nutritional quality and functional properties of gluten-free foods. Therefore, this study aimed to characterize and compare the structural, elemental, thermal, and energetic properties of flours from black and yellow morphotypes of *T. tuberosum*. By integrating advanced techniques such as carbon, hydrogen, nitrogen, and sulfur (CHNS) elemental analysis, scanning electron microscopy, differential scanning calorimetry, thermogravimetric analysis, and bomb calorimetry, this study contributes novel data to the functional and technological understanding of this underutilized Andean crop.

## 2. Results and Discussion

### 2.1. Approximate Organic Composition of Flours

The elemental and proximate composition of the black and yellow mashua flours are summarized in Table 1. The carbon, hydrogen, nitrogen, and sulfur contents determined by CHNS analysis accounted for nearly 50% of the total dry mass, with oxygen content estimated by the difference. Based on these values, the empirical formulas of the black and yellow flours were calculated as CH_1.74_O_0.91_N_0.06_S_0.005_ and CH_1.78_O_0.92_N_0.05_S_0.005_, respectively, indicating similar elemental profiles with slightly higher nitrogen content in the black morphotype. Protein content, estimated from nitrogen concentration (N × 6.25), was higher in black flour (17.6%) compared to yellow flour (14.8%) at a statistically significant level (*p* < 0.001), placing both at the upper end of the ranges reported for *Tropaeolum tuberosum* genotypes in previous studies such as that of Colona et al. [4], who reported that the protein content is higher in the purple genotype (7.41 to 11.72 g/100 g) than in yellow (6.96 to 9.98 g/100 g) and yellow-purple genotypes (7.15 to 7.95 g/100 g). In contrast, Castañeta et al. [15] reported no significant differences in total protein content between fresh samples of yellow and purple mashua. Similarly, the fat content was greater in black mashua (8.0%) than in yellow mashua (6.7%) at a statistically significant level (*p* < 0.001), whereas the moisture and ash values remained within the expected ranges for Andean tuber flours [5]. These findings support the nutritional richness of both flour types and suggest that black mashua flour, in particular, could be a more valuable ingredient in formulations requiring high protein and fat contents. However, the high fat content in flours is associated with enzymatic oxidation, which generates free fatty acids and can lead to rancidity [16]. Although no specific shelf-life study has been conducted for mashua flour, studies on similar tuber flours indicate good stability when stored under optimal conditions (low humidity, cool temperatures, protection from light), combined with appropriate packaging materials [17], and with suitable pretreatment techniques applied to the raw material [18]. The presence of sulfur, although low, confirms the contribution of sulfur-containing phytochemicals such as glucosinolates and supports the observed biofunctional potential of this Andean crop [19,20].

Additionally, the nitrogen content (2.36–2.81%) observed in mashua flours corresponded to crude protein levels higher (14.8–17.6%) than those typically reported for wheat flour (10.82–12.75% protein) [21], rice flour (7.28 ± 0.53 %) [22], and maize flour (9.15–10.12%) [23], and considerably higher than potato starch (1.06–1.16%) [24]. The relatively elevated protein and mineral contents in mashua flour, combined with the absence of gluten, suggest the potential to enhance the nutritional profile of gluten-free bakery and pasta formulations. 

**Table 1:** Elemental and proximate composition (dry basis) of black and yellow *Tropaeolum tuberosum* flours, and comparison with literature values.

Parameter	Black Mashua	Yellow Mashua	Literature Range	References
Elemental composition (% m/m)				
Carbon (C)	41.06 ± 0.04	40.96 ± 0.02	—	—
Hydrogen (H)	6.01 ± 0.04	6.11 ± 0.04	—	—
Nitrogen (N)	2.81 ± 0.18	2.36 ± 0.16	—	—
Sulfur (S)	0.56 ± 0.08	0.55 ± 0.02	4.9–54.2 *	[25]
Estimated Oxygen + others (%)	49.58 ± 0.02	50.03 ± 0.22	—	—
Empirical formula	CH_1.74_O_0.91_N_0.06_S_0.005_	CH_1.78_O_0.92_N_0.05_S_0.005_	—	—
Proximate composition (% m/m)				
Moisture	5.36 ± 0.02	6.35 ± 0.04	4.4–18.9	[5,26,27]
Crude protein (N × 6.25)	17.6 ± 1.1 ^a^	14.8 ± 1.1 ^b^	6.9–15.7	[4,15,28]
Crude fat	8.0 ± 0.5 ^a^	6.7 ± 0.5 ^b^	0.6–6.2	[4,5,6]
Ash (TGA)	5.6	5.1	3.6–7.9	[4,15,27]
Ash (Calorimetry residue)	4.3	3.6	—	—

Values with different superscript letters in the same row are significantly different between morphotypes (*p* < 0.05, Sidak–Bonferroni multiple *t*-test). * Literature data on glucosinolates in mashua (4.9–54.2 μmol/g) are reported instead of elemental sulfur [25], since sulfur occurs mainly in glucosinolates. Note: Elemental composition determined by CHNS analysis. Moisture calculated from mass loss during drying; protein estimated from nitrogen content using N × 6.25; fat content estimated based on empirical protein/fat ratio; ash determined from thermogravimetric analysis (TGA) and confirmed by inorganic residue post-combustion. Values in the literature include a range of Peruvian and Ecuadorian mashua genotypes [4,5,6,25,26,27,28,29].

### 2.2. Content of Macro- and Microlements

The concentrations of macro- and microelements in the black and yellow mashua flours are shown in Table 2. Both morphotypes exhibited high levels of K (~24,000 mg/kg) and Mg (~1400 mg/kg), consistent with previous reports on Andean tubers [5,30,31]. These values suggest that mashua flour is an excellent source of essential macro- and microelements. Notably, black mashua flour had almost twice the iron content (236 mg/kg) of yellow flour (127 mg/kg), whereas calcium and sodium levels were significantly lower in the black morphotype. This inverse trend may be linked to the varietal differences in mineral uptake and storage. Phosphorus concentrations were slightly higher in black mashua (4317 mg/kg) than in yellow mashua (4132 mg/kg), which is similar to values previously reported in the literature [27,30]. 

The concentrations of trace elements such as manganese, zinc, and copper were relatively similar between both morphotypes, although all were within or slightly below the ranges reported for other *Tropaeolum tuberosum* accessions. When compared to other gluten-free flours, such as those from Andean pseudocereals (quinoa, kiwicha, kañiwa) [14], mashua flours showed similar levels of Ca, Mg, P, Mn, Zn, and Na, and Fe levels comparable to those of kañiwa, and markedly high K concentrations of up to five times greater than those found in pseudocereal flours. The low sodium content and high potassium levels in both flours may be particularly advantageous for individuals with hypertension or cardiovascular conditions, thereby reinforcing the potential of mashua as a functional food.

**Table 2:** Macro- and microelement contents (mg/kg dry matter) of black and yellow *Tropaeolum tuberosum* flours, and comparison with values in the literature.

Element	Black Mashua	Yellow Mashua	Literature Range	References
Calcium (Ca)	457	710	347.8–900	[4,31,32]
Potassium (K)	24,869	24,425	17,234–32,500	[4,31,32]
Magnesium (Mg)	1474	1365	1100–1640	[30,32]
Sodium (Na)	60	99	160-440	[27,31,32]
Phosphorus (P)	4317	4132	1145.6–3200	[29,31,32]
Iron (Fe)	236	127	28–86	[31,32]
Manganese (Mn)	11	10	7–15.5	[4,31,33]
Zinc (Zn)	29	23	20.6–119.9	[27,31,32]
Copper (Cu)	3.1	2.4	9–19.6	[27,31]

Note: Elemental concentrations were determined using ICP-OES. The results were expressed as mg of element per kg of dry flour. The values in the literature correspond to various Andean tuber reports from Peru and Ecuador [4,27,31,32,33].

### 2.3. Morphological Characterization

**Figure 1:**
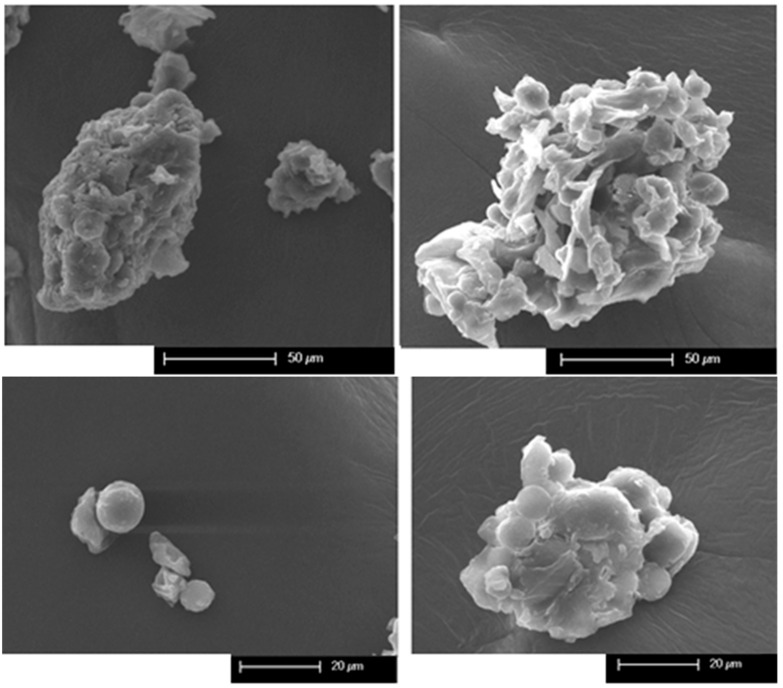
SEM of mashua (*Tropaeolum tuberosum*) flours from two morphotypes. Left column: black morphotype; right column: yellow morphotype. Upper images show the general matrix structure, including fibrous components and amorphous material. Lower images highlight the morphology of starch granules, which appear predominantly spherical to ellipsoidal, with average diameters of ~10 µm.

Scanning electron microscopy (SEM) revealed similar morphological characteristics for both mashua flour (black and yellow) morphotypes (Figure 1). At low magnification, the flour matrix displayed a heterogeneous structure composed of fibrous material and disaggregated cellular components. At higher magnification, abundant starch granules with predominantly round to ellipsoidal shapes and an average diameter of approximately 10 µm were observed, consistent with the size range (length: 4.39–16.29 µm, diameter: 4.07–13.09) reported for *Tropaeolum tuberosum* starches in previous studies [6]. According to the literature, the microscopic characteristics observed in the size and shape of starch granules are fundamental for determining their technological properties. In cassava, the starch granules, predominantly spherical and oval with sizes ranging from 4 µm to 23 µm, display significant variability in amylose content, in-vitro digestibility, and viscosity characteristics [34]. These differences influence their hydration and textural behavior, and, consequently, their performance in food applications. Other studies reported that tacca starch exhibited irregularly shaped, tiny granules with polyhedral edges, whereas maize starch showed round to oval granules with a relative lack of asperity. Tacca starch (*Tacca leontopetaloides*) has very small granules with a mean particle size of 2.64 μm, whereas maize starch consists of medium granules with a mean particle size of 14.36 μm. Tacca starch shows a monomodal narrow distribution with most particles between 0.8 μm and 6.0 μm, whereas maize starch has a broad size distribution ranging from about 8.0 μm to 32.0 μm [35]. This characteristic influences various physicochemical, functional, and nutritional properties: large granules can produce high paste viscosity, while small granules offer greater digestibility [36,37].

### 2.4. Thermal and Energetic Characterization

**Figure 2:**
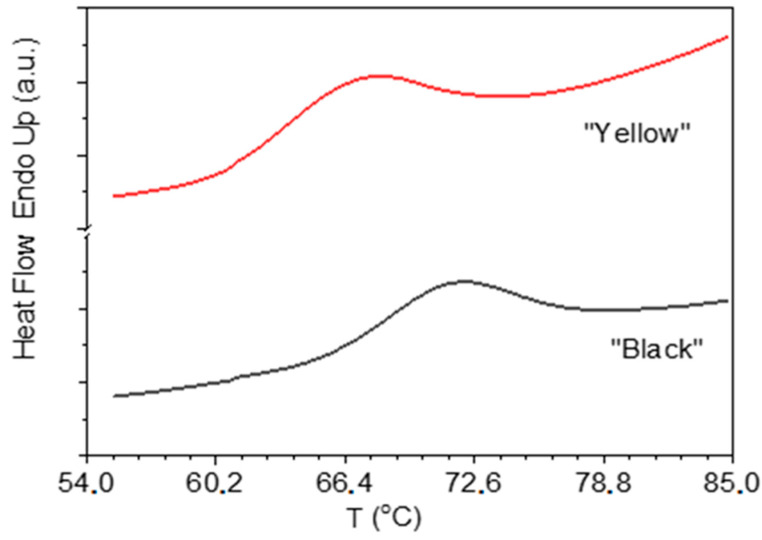
Differential Scanning Calorimetry (DSC) thermograms of mashua (*Tropaeolum tuberosum*) flours. Upper curve: yellow morphotype; lower curve: black morphotype. Both samples exhibit a characteristic endothermic transition corresponding to starch gelatinization. The black flour shows a shift toward higher onset (Ti), peak (Tmax), and conclusion (Tf) temperatures compared to the yellow flour, indicating greater thermal stability. Measurements were performed with ~7 mg of flour in stainless steel pans under nitrogen atmosphere, with a heating rate of 10 °C/min over the range 54–85 °C.

The thermal behavior of the black and yellow mashua flours was further explored using DSC analysis. Representative thermograms are shown in Figure 2, and the corresponding thermal and energetic parameters are summarized in Table 3. The DSC curves exhibit typical endothermic transitions associated with starch gelatinization, beginning at approximately 61.2 °C for yellow mashua and 65.5 °C for black mashua. Black flour consistently showed higher onset (T_i_), peak (T_max_), and conclusion (T_f_) temperatures than yellow flour (*p* < 0.001), indicating greater thermal stability of its starch matrix. These shifts could be attributed to differences in starch structure, particularly in amylopectin architecture and the presence of other flour constituents such as proteins, polyphenols, and lipids, which are known to influence gelatinization behavior [38,39]. It is important to highlight the presence of antioxidants, especially polyphenols, which interact in a complex manner during starch (flour) gelatinization and significantly influence its thermal and textural properties. The interaction between antioxidants and starch-chain molecules is a well-established and actively researched area in food science [40,41,42] which occurs primarily through hydrogen bonding and the formation of complexes that reinforce and stabilize the starch granule structure and leads to an increase in the gelatinization temperature, requiring more energy to disrupt this structure and thus allow water penetration. In this context, the higher gelatinization temperatures determined in black compared to yellow mashua indicate the presence of weaker starch–antioxidant interactions in the latter. Despite the differences in gelatinization temperatures, both flours exhibited similar gelatinization enthalpies (ΔH_gel_: 2.0 ± 0.2 J/g for yellow mashua and 2.2 ± 0.2 J/g for black mashua), suggesting that the total energy required for gelatinization is comparable between the two morphotypes. The low enthalpy values, also observed in flours from other Andean tubers [5,26,43], would be due to the presence of antioxidants that partially disrupted the starch structure or limited water uptake.

On the other hand, in some studies carried out in other starches such as tacca starch (*Tacca leontopetaloides*), the gelatinization parameters of tacca starch were found to be lower than those of maize starch. The onset, peak, and conclusion temperatures for tacca starch were 65.57 °C, 68.56 °C, and 73.10 °C, respectively, while for maize starch they were 67.30 °C, 70.97 °C, and 76.25 °C. The enthalpy of gelatinization for tacca starch (3.49 J/g) is considerably lower than that of maize starch (7.01 J/g) [35]. These results indicate that the associative forces that stabilize the granule structure in tacca starch are weaker than those in maize starch.

**Table 3:** Gelatinization thermal and energetic parameters of black and yellow *Tropaeolum tuberosum* flours.

Parameter	Black Mashua	Yellow Mashua	Literature Range	References
Onset temperature (T_i_, °C)	65.5 ± 1.0 ^a^	61.2 ± 1.2 ^b^	51.85–69.86	[3,5,6,26,43]
Peak temperature (T_max_, °C)	72.0 ± 1.6 ^a^	67.8 ± 2.2 ^b^	54.9–73.81	[3,5,6,26,43]
Conclusion temperature (T_f_, °C)	79.7 ± 3.4 ^a^	75.0 ± 4.1 ^b^	60.7–78.29	[3,5,6,26,43]
Gelatinization enthalpy (ΔH_ge_l, J/g)	2.2 ± 0.2	2.0 ± 0.2	1.2–14.3	[3,5,6,26,43]

Values with different superscript letters in the same row are significantly different between morphotypes (*p* < 0.05, Sidak–Bonferroni multiple *t*-test). Note: Thermal parameters were determined by DSC (Differential Scanning Calorimetry). Values represent the mean ± standard deviations of four replicate measurements. The literature includes values from similar Andean tubers and starch sources.

**Figure 3:**
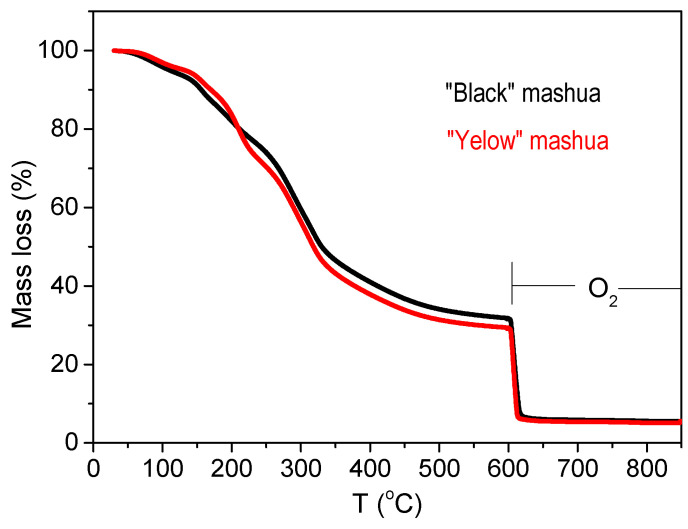
Thermogravimetric analysis (TGA) of mashua (*Tropaeolum tuberosum*) flours. (Black color line) Black morphotype; (red color line) Yellow morphotype. Curves represent mass loss (%) as a function of temperature. Three main decomposition stages are observed: moisture loss (~30–120 °C), decomposition of organic compounds (150–350 °C), and carbonization (350–600 °C). Final combustion under oxygen (600–850 °C) led to stable inorganic residues.

On the other hand, Figure 3 shows the thermogravimetric analysis (TGA) curves of both mashua flours under controlled heating conditions. Additional TGA profiles are provided in the Supplementary Materials (Figure S1), including the thermal degradation under nitrogen and subsequent combustion under oxygen. The weight loss profiles exhibited three major decomposition phases. The first stage, observed between 30 and 120 °C, corresponds to moisture evaporation, and aligns well with the estimated moisture contents listed in Table 1 (approximately 5–6%). The second phase, spanning 150–350 °C, was associated with the thermal decomposition of organic constituents—particularly proteins, simple sugars, and polyphenolic compounds. This phase accounted for the largest mass loss in both morphotypes. The final stage, extending from ~350 to 600 °C, represents the carbonization and degradation of more stable macromolecules such as starch and fiber. At the end of this phase, a residual mass of approximately 30% remained, indicating the relative thermal resistance of the flour. 

Complete combustion in the presence of oxygen occurred above 600 °C, with the total mineral residue (ash) stabilizing at 5.6% for black flour and 5.1% for yellow flour. These values are consistent with the sum of the elemental contents (macro- and microelements) presented in Table 2, confirming the accuracy of both methods. The TGA profiles demonstrate that mashua flours possess good thermal stability and predictable degradation behavior, making them suitable for applications involving moderate to high heat processing. The slightly higher residual mass in black flour is in agreement with its higher content of thermally stable compounds such as proteins and minerals.

The detailed results of the combustion calorimetry experiments for the black and yellow mashua flours are presented in Table 4. Additional parameters and replicate measurements are available in the Supplementary Materials (Table S1). All combustion processes were complete, with no detectable carbon residues, CO, or soot, and the final ash was collected and quantified. The net calorific value (q_NCV_) was significantly higher (*p* < 0.001) in black mashua flour (4157 ± 22 kcal/kg) than in yellow mashua flour (4022 ± 19 kcal/kg), confirming the greater energetic potential of the black morphotype. The calculated standard massic energy of combustion (−Δ_c_u°) was 17.39 ± 0.09 kJ/g for black flour and −16.83 ± 0.08 kJ/g for yellow flour, values that are consistent with energy densities reported for other carbohydrate-rich plant matrices. These values were notably higher than those reported by Salazar et al. [5] using the estimated caloric content, and aligned more closely with those obtained via experimental calorimetry in studies on Andean tubers [27,31]. The ash content derived from the combustion residues (4.3% for black and 3.6% for yellow) correlated well with the values obtained from the TGA and elemental analysis (Table 1 and Table 2), reinforcing the analytical consistency of the thermal and energetic profiles. Additionally, the slightly higher q_NCV_ of black mashua flour may be explained by its greater content of nitrogenous and phenolic compounds, such as polyphenols and flavonoids, which contribute to the higher combustion energy values. These findings highlight the potential application of mashua flours, particularly those from the black morphotype, in the formulation of high-energy functional foods or in contexts requiring enhanced caloric density, such as sports nutrition or dietary support for undernourished populations.

The main limitations of this study were that it did not evaluate the effect of storage on the physicochemical properties of mashua flours or the instrumental color measurements during drying, milling and storage. Further research should assess how key parameters (moisture, lipid oxidation, starch gelatinization, color) evolve under controlled storage conditions to determine the shelf life and optimize packaging for gluten-free product development.

## 3. Materials and Methods

### 3.1. Collection of Plant Species

**Figure 4:**
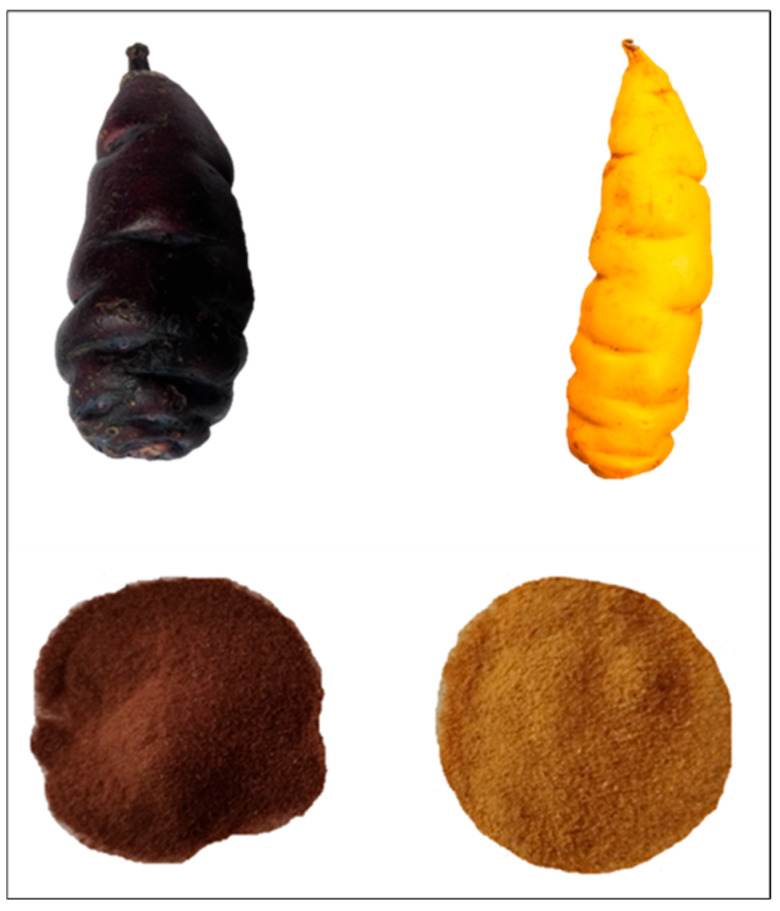
Visual comparison of the two *Tropaeolum tuberosum* (mashua) morphotypes studied. Upper panel: whole tubers of the black (left) and yellow (right) morphotypes collected from Iquicha, Ayacucho, Peru. Lower panel: corresponding flours obtained after slicing, drying, and milling of the tubers. Differences in tuber pigmentation are evident and visually maintained in the flour color, reflecting the distinct phytochemical composition associated with each morphotype.

Two morphotypes of mashua tubers (“black” and “yellow”, see Figure 4) were collected from Iquicha, Ayacucho, Peru, at an altitude of 3802 m a.s.l. (13°23′13.5″ S, 74°08′39.6″ W). Morphological characterization was performed under controlled conditions at the Cellular and Molecular Biology Laboratory of the Universidad Nacional San Cristóbal de Huamanga (UNSCH).

### 3.2. Obtention of Mashua Flour

Fresh mashua tubers (yellow and black morphotypes) were first washed, sliced, blanched, dehydrated, and milled according to the established protocol. For each morphotype, three independent batches of fresh tubers (3.0 kg per batch) were processed separately. The dehydration process was carried out under controlled conditions (65 ± 2 °C for 24 h), followed by milling at 1800 rpm for 2 min (double pass) and sieving through a No. 100 mesh. This procedure yielded approximately 300 g of fine flour per batch, corresponding to a 10% yield on a dry weight basis. 

### 3.3. Elemental Analysis

The elemental composition of the two morphotypes of mashua flour, specifically carbon (C), hydrogen (H), nitrogen (N), and sulfur (S), was determined using a LECO CHNS-932 elemental analyzer (LECO Corporation, St. Joseph, MI, USA). Approximately 1–3 mg of finely powdered and oven-dried material was accurately weighed into pre-cleaned tin capsules using a microbalance with ± 0.01 mg precision (UXM2 Mettler-Toledo, Greifensee, Switzerland). Samples were combusted in a stream of pure oxygen at high temperatures (~950–1000 °C), and the resulting gases were measured as follows: C, H, and S were quantified via infrared detection (IQM-CSIC, Madrid, Spain), while N was determined using a thermal conductivity detector (IQM-CSIC-Madrid, Spain). The instrument was calibrated with a certified organic standard (CENQUIOR-CSIC, Madrid, Spain), and blank samples were used to control the baseline drift. Each measurement was performed in triplicate, and the results were reported as the mean ± standard deviation. The oxygen content of each sample was calculated by subtracting the sum of the percentages of C, H, N, and S from 100. Empirical molecular formulas were established based on elemental percentages using normalized atomic ratios [44]. Crude protein content was determined using the Kjeldahl method, and a nitrogen-to-protein conversion factor of 6.25 was applied. Although this universal factor may slightly overestimate the true protein content in some plant matrices, no specific conversion factor has been reported for *Tropaeolum tuberosum*. Therefore, this method has been validated in collaborative AOAC studies of cereal and oilseed flours [45].

### 3.4. Determination of Macro- and Microelements

The content of macro- and microelements (Ca, K, Mg, Na, P, Fe, Mn, Zn, and Cu) was quantified using Inductively Coupled Plasma–Optical Emission Spectroscopy ICP-OES (PerkinElmer, Waltham, MA, USA) [46]. Prior to analysis, the flour samples were digested using nitric acid and perchloric acid (at a ratio of 4:1 *v*/*v* in a microwave-assisted digestion system (ICA-CSIC, Madrid, Spain). The complete digestion lasted approximately 45 min, reaching a temperature of 200 °C, without pressure control. After digestion, the solutions were filtered (0.45 μm) and brought to final volumes of 25, 50, and 100 mL. Quantification was performed by external calibration with multi-element certified reference standards (ICP Multi-Element Standard Solution IV, Certipur®, Merck, Darmstadt, Germany), and all analyses were conducted in triplicate.

### 3.5. Morphological Characterization by Scanning Electron Microscopy (SEM)

The surface morphology and microstructure of the flour granules were examined using a Philips XL-30 ESEM scanning electron microscope (Philips, Eindhoven, The Netherlands). The samples were mounted on aluminum stubs using carbon tape and sputter-coated with a thin layer of gold (5–20 nm) to ensure conductivity [47]. The SEM parameters (acceleration voltages, beam and aperture currents) were adjusted to achieve a good balance between signal and resolution. The secondary electron (SE) detector mode was used to analyze the topography and surface details. The SEs were focused on adjusting the brightness and contrast. Imaging was conducted at various magnifications under high vacuum.

### 3.6. Thermal and Energetic Characterization

#### 3.6.1. Differential Scanning Calorimetry (DSC)

Thermal transitions related to starch gelatinization were analyzed by a DSC 822e instrument (Mettler-Toledo, Greifensee, Switzerland) [47]. Approximately 7 mg of flour was weighed into stainless steel crucibles and mixed with distilled water (water-to-sample ratio: 3:1, *v*/*w*). After equilibrating at room temperature for 12 h, the samples were scanned from 54 to 85 °C at a heating rate of 10 °C·min^−1^ under a nitrogen atmosphere. The onset (T_i_), peak (T_max_), and conclusion (T_f_) temperatures, along with the enthalpy of gelatinization (ΔH_gel_), were recorded and calculated using STARe software, version 16.20 (Mettler-Toledo, Greifensee, Switzerland). All experiments were conducted in quadruplicate.

#### 3.6.2. Thermogravimetric Analysis (TGA)

Thermal stability and degradation patterns were studied by thermogravimetric analysis using a Mettler-Toledo TGA 2 instrument (Mettler-Toledo, Greifensee, Switzerland). Approximately 10 mg of sample was heated from 30 to 600 °C under nitrogen, and then from 600 to 850 °C under an oxygen atmosphere, both at a constant heating rate of 10 °C·min^−1^. Mass loss events were recorded as a function of temperature to determine moisture content, organic matter decomposition, and final ash residue [48].

#### 3.6.3. Combustion Calorimetry

The energy content of the mashua flours was determined by isoperibolic combustion calorimetry using a custom-built system based on the established protocols [14,49]. The calorimeter (IQF-CSIC, Madrid, Spain) consisted of a static bomb housed within an internal vessel, which was surrounded by a thermostatically controlled water jacket at 25.2 °C. The internal temperature of the system was initially set at 23.5 °C. As the device is non-adiabatic, heat exchange between the bomb and the surroundings was corrected using Newtonian heat transfer principles, by extrapolating the baseline drift before and after combustion to the point of maximum temperature, following the methodology described by Hubbard et al. [50].

For each experiment, flour (~0.5 g) was compressed into pellets and combusted in a static oxygen atmosphere (3.04 MPa) inside a stainless-steel bomb. Benzoic acid (0.26–0.29 g, −26,434 ± 3 J/g), was used as an auxiliary standard to ensure complete combustion, together with a cotton thread (~0.004 g) as the ignition fuse. The increase in temperature was monitored using a high-precision platinum resistance thermometer (Model F300, A.S.L. Ltd., Guildford, UK), with an accuracy of ± 0.0001 °C. The calorimeter’s energy equivalent (ε) was calibrated using five replicate combustions of benzoic acid (NIST 39j reference standard, National Institute of Standards and Technology, Gaithersburg, MD, USA; −26,434 ± 3 J/g), resulting in a value of 14,249.6 ± 5.2 J/°C. 

The standard massic energy of combustion (Δ_c_u°) of each flour sample was calculated using the following equation:

Δ_c_u° = [ε_calor ΔT_ad + ε_cont ΔT_ad + ΔU_HNO_3_ + ΔU_corr + m′·Δ_c_u°(benzoic) + m″·Δ_c_u°(cotton)]/m (1)

Δ_c_u°: Standard massic energy of combustion of the sample (J/g); ε_calor: Energy equivalent of the calorimetric system excluding bomb contents (J/°C); ε_cont: Energy equivalent of the bomb contents (J/°C); ΔT_ad: Corrected temperature rise during combustion (°C); ΔU_HNO_3_: Energy correction due to formation of nitric acid (J); ΔU_corr: Washburn correction to standard state (J); m: Mass of the flour sample (g); m′: Mass of benzoic acid used as combustion standard (g); Δ_c_u°(benzoic): Standard energy of combustion of benzoic acid (−26,434 ± 3 J/g); m″: Mass of cotton thread used as ignition fuse (g); Δ_c_u°(cotton): standard energy of combustion of cotton (−17,410 ± 37 J/g).

After each combustion, the bomb was opened, and the residual ash was collected and weighed. The acidity of the remaining aqueous phase was titrated with 0.1 N NaOH to determine the formation of nitric acid. To ensure the validity of the experiments, the presence of CO or incomplete combustion was verified using Dräger tubes (Draeger Inc. Houston, TX, USA). Finally, the net calorific value (q_NCV_) was derived from the absolute value of the average Δ_c_u° and was expressed in kcal/kg and kcal per 100 g of dry flour. Uncertainty propagation was addressed by combining the contributions of the sample mass, temperature increase, and ε according to conventional error propagation rules. All experiments were performed in quadruplicate, with an experimental uncertainty below 0.5%.

### 3.7. Statistical Analysis

Data were expressed as mean ± standard deviation (SD). For elemental composition, thermogravimetric analysis, and combustion calorimetry analysis of *Tropaeolum tuberosum* flours, comparisons between black and yellow morphotypes were performed using multiple *t*-tests with Sidak–Bonferroni correction to control the family-wise error rate. Statistical significance was set at *p* < 0.05. Statistical analyses were performed using the GraphPad Prism software, version 6.0 (GraphPad Software Inc., San Diego, CA, USA). 

## 4. Conclusions

This study provided a comprehensive physicochemical, nutritional, and thermo-energetic characterization of two *Tropaeolum tuberosum* morphotypes (black and yellow) flour from Peru. Elemental and proximate composition analyses revealed that both morphotypes are rich in protein, minerals, and lipids, with significant differences in crude protein and fat content, and particularly high potassium levels compared to other gluten-free flours. SEM imaging confirmed well-defined starch granules with morphologies typical of tuber starches, supporting the DSC findings of relatively low gelatinization temperatures. Thermogravimetric and calorimetric data demonstrated high energy values and thermal stability above 150 °C, indicating suitability for a wide range of processing conditions. Overall, these results not only differentiate the two morphotypes but also position mashua flour as a promising gluten-free raw material with functional and nutritional advantages, suitable for the development of high-energy functional foods and potential fortification of gluten-free bakery and pasta products. 

## Figures and Tables

**Table 4:** Representative combustion calorimetry results of black and yellow *Tropaeolum tuberosum* flours.

Parameter	Black Mashua	Yellow Mashua	Units
Flour mass (m)	0.5071	0.5021	g
Benzoic acid mass (m′)	0.2812	0.2910	g
Cotton mass (m″)	0.0040	0.0032	g
Inorganic residue (m‴)	0.0213	0.0173	g
Corrected temperature rise (T_ad_)	1.1187	1.1151	°C
Net calorific value (q_NCV_)	4157 ± 22 ^a^	4022 ± 19 ^b^	Kcal/kg
q_NCV_ (100 g basis)	415.7 ± 2.2	402.2 ± 1.9	Kcal/100 g
Standard combustion energy (−Δ_c_u°)	17.39 ± 0.09	16.83 ± 0.08	kJ/g
Ash residue	4.3	3.6	%

Different superscript letters within a row indicate significant differences between morphotypes (*p* < 0.05, Sidak–Bonferroni multiple *t*-test). Note: Values represent the typical results of isoperibolic combustion calorimetry. q_NCV_: net calorific value. −Δ_c_u°: Standard massic energy of combustion. Residual ash was determined after post-combustion. The results are the mean of four replicates with uncertainties below 0.5%.

## Data Availability

The datasets generated during and/or analyzed during the current study are available from the corresponding author upon reasonable request.

## Supplementary Materials

The following supporting information can be downloaded at: https://www.mdpi.com/article/10.3390/molecules30173560/s1, Figure S1: Thermogravimetric analysis (TGA) profiles of black and yellow morphotypes of *Tropaeolum tuberosum* flours. Samples were heated from 30 to 600 °C under nitrogen, followed by combustion from 600 to 850 °C under oxygen. The curves show mass loss (%) versus temperature, indicating moisture evaporation, decomposition of organic matter, and final carbonization/combustion. Slightly higher residual mass was observed in black flour; Table S1: Detailed combustion calorimetry parameters of black and yellow *Tropaeolum tuberosum* flours using benzoic acid as standard and cotton as ignition fuse. The symbols were defined according to Hubbard et al. [50].
