# Supplementary material for: Nutritional, Thermal, and Energetic Characterization of Two Morphotypes of Andean Mashua (Tropaeolum tuberosum Ruiz & Pavón) Flours from Peru
Source: Molecules. 2025 Aug 30;30(17):3560. doi: 10.3390/molecules30173560 (PMC12430478; doi:10.3390/molecules30173560)
Supplement: Supplementary file 1 [file molecules-30-03560-s001.zip › molecules-3785028-supplementary.pdf]

Supplementary material

Article

Comparative Thermo-Energetic and Nutritional Characterization of Two Andean Mashua (*Tropaeolum tuberosum* Ruiz & Pavón) Flour Morphotypes from Peru

Gilmar Peña-Rojas [1,*], Vidalina Andía-Ayme [2], Alberto Fernández-Torres [3], Juan Z. Dávalos-Prado [4], and Oscar Herrera-Calderon [5,*]

[1] Laboratorio de Biología Celular y Molecular, Universidad Nacional de San Cristóbal de Huamanga, Ayacucho 05003, Perú;

[2] Laboratory of Food Microbiology, Biological Sciences Faculty, Universidad Nacional de San Cristóbal de Huamanga, Ayacucho 05003, Peru; vidalina.andia@unsch.edu.pe

[3] Instituto Ciencia y Tecnología de Polímeros-CSIC, Juan de la Cierva 3, 28006 Madrid, Spain; a.f.torres@csic.es

[4] Instituto de Química-Física "Blas Cabrera"-CSIC, Serrano119, 28006 Madrid, Spain; jdavalos@iqf.csic.es

[5] Department of Pharmacology, Bromatology and Toxicology, Faculty of Pharmacy and Biochemistry, Universidad Nacional Mayor de San Marcos, Lima 15001, Peru

* Correspondence: gilmar.pena@unsch.edu.pe; oherreraca@unmsm.edu.pe; Tel.: +51 956 550 510

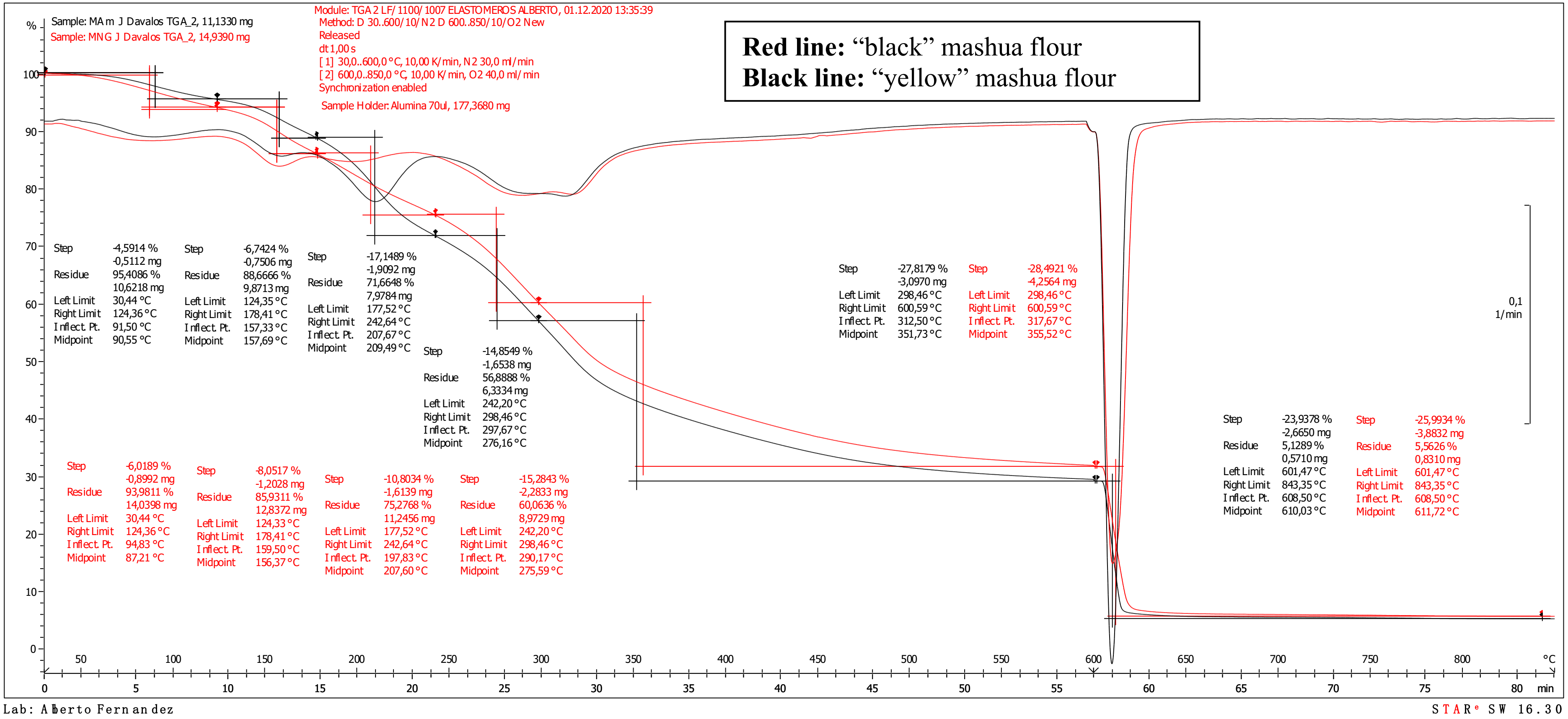

Figure S1. Thermogravimetric analysis (TGA) profiles of black and yellow morphotypes of *Tropaeolum tuberosum* flours. Samples were heated from 30 to 600 °C under nitrogen, followed by combustion from 600 to 850 °C under oxygen. The curves show mass loss (%) versus temperature, indicating moisture evaporation, decomposition of organic matter, and final carbonization/combustion. Slightly higher residual mass was observed in black flour.

Table S1. Detailed combustion calorimetry parameters of black and yellow *Tropaeolum tuberosum* flours using benzoic acid as standard and cotton as ignition fuse. The symbols were defined according to Hubbard *et al.**

	"black" mashua flour				"yellow" mashua flour			
m (compound) /g [a]	0.4858	0.4954	0.4933	0.4866	0.4920	0.4869	0.4844	0.4848
m´(benzoic acid) /g [b]	0.2812	0.2972	0.3417	0.3241	0.2684	0.3152	0.3030	0.2910
m´´(fuse) /g [c]	0.0040	0.0040	0.0036	0.0034	0.0036	0.0036	0.0040	0.0032
ΔT_{ad} / K [d]	1.1187	1.1592	1.2450	1.1971	1.0845	1.1605	1.1415	1.1151
ε(calor)(-ΔT_{ad}) / kJ [e]	-15.943	-16.520	-17.742	-17.061	-15.455	-16.539	-16.268	-15.892
ε(cont.)(-ΔT_{ad}) / kJ [f]	-0.018	-0.019	-0.021	-0.020	-0.018	-0.019	-0.019	-0.018
$\Delta U(HNO_3)$/ kJ [g]	0.008	0.009	0.008	0.009	0.008	0.007	0.003	0.003
ΔU(corr. to std. states)/kJ [h]	0.011	0.012	0.013	0.012	0.011	0.012	0.012	0.012
- m′ $\Delta_c u°$(benzoic) / kJ [i]	7.434	7.857	9.033	8.568	7.094	8.332	8.010	7.693
- m″ $\Delta_c u°$(fuse) / kJ [j]	0.070	0.069	0.062	0.058	0.063	0.063	0.070	0.055
$\Delta_c u°$(compound) / kJ g^{-1} [k]	-17.37	-17.34	-17.53	-17.33	-16.86	-16.73	-16.91	-16.81
⟨$\Delta_c u°$(298.15 K)⟩/ kJ·g^{-1} [l]	-17.39 ± 0.09				-16.83 ± 0.08			
q_{NCV} /kJ g^{-1} [m]	17.39 ± 0.09				16.83 ± 0.08			
q_{NCV} /kcal kg^{-1}	4157 ± 22				4022 ± 19			
q_{NCV} /kcal $(100g)^{-1}$ [n]	415.7 ± 2.2				402.2 ± 1.9			
m_{res} /g [o]	0.0213	0.0232	0.0249	0.0192	0.0198	0.0181	0.0173	0.0173
m_{res}%.[p]	7.1				6.2			

Summary of calorimetric combustion experiments conducted for black and yellow mashua (*Tropaeolum tuberosum*) flours. Experiments were performed in oxygen atmosphere using a static isoperibolic bomb calorimeter. Each sample was combusted with benzoic acid and an ignition cotton fuse. The final residual ash was also recorded. Results are based on four replicates. Notation and calculations are based on the protocol of Hubbard et al. (1956). [a] Corrected mass, taking into account combustion residues. [b] Mass of benzoic acid used as combustion auxiliary. [c] Mass of fuse (cotton). [d] Corrected temperature rise. [e] Total energy released in the heat form. ε(calor) is the energy equivalent of the whole system excluding the heat capacity of the bomb. [f] ε(cont.) energy equivalent of the contents of the

bomb. [g] Energy of formation of nitric acid. [h] Standard state correction. [i] Energy released by benzoic acid combustion. [j] Energy released by fuse combustion. [k] Standard massic energy of combustion, $\Delta_c u^o$(sample) = Σ[(e) to (j)]. [l] Average value of $\Delta_c u^o$ and its standard uncertainty for four experiments. It includes mass correction according to the inorganic residues from combustion. [m] Net calorific value associated to the absolute value of standard massic energy of combustion. [n] q_{NCV} in kcal by 100 g of dry sample. [o] combustion or inorganic residue. [p] Average percentage of combustion residues.

* Hubbard, W.N.; Scott, D.W.; Waddington, G. Experimental Thermochemistry; Rossini, F.D., Ed.; Interscience: New York, NY, USA, 1956; Chapter 5.
